# Fiber Cell-Specific Expression of the VP16-Fused Ethylene Response Factor 41 Protein Increases Biomass Yield and Alters Lignin Composition

**DOI:** 10.3389/fpls.2021.654655

**Published:** 2021-04-30

**Authors:** Miyuki T. Nakata, Shingo Sakamoto, Nuoendagula, Shinya Kajita, Nobutaka Mitsuda

**Affiliations:** ^1^Plant Gene Regulation Research Group, Bioproduction Research Institute, National Institute of Advanced Industrial Science and Technology (AIST), Tsukuba, Japan; ^2^Smart CO_2_ Utilization Research Team, Global Zero Emission Research Center, National Institute of Advanced Industrial Science and Technology (AIST), Tsukuba, Japan; ^3^Graduate School of Bio-Applications and Systems Engineering (BASE), Tokyo University of Agriculture and Technology (TUAT), Koganei, Japan

**Keywords:** *Arabidopsis thaliana*, ERFIIId/e transcription factors, primary cell wall, fiber cell, lignin, syringyl (S)/guaiacyl (G) ratio, ferulate 5-hydroxylase1 (F5H1)

## Abstract

*Arabidopsis thaliana* transcription factors belonging to the ERFIIId and ERFIIIe subclade (ERFIIId/e) of the APETALA 2/ethylene response factor (AP2/ERF) family enhance primary cell wall (PCW) formation. These transcription factors activate expression of genes encoding PCW-type cellulose synthase (CESA) subunits and other genes for PCW biosynthesis. In this study, we show that fiber-specific expression of ERF035-VP16 and ERF041-VP16, which are VP16-fused proteins of ERFIIId/e members, promote cell wall thickening in a wild-type background with a concomitant increase of alcohol insoluble residues (cell wall content) per fresh weight (FW) and monosaccharides related to the PCW without affecting plant growth. Furthermore, in the *ERF041-VP16* lines, the total amount of lignin and the syringyl (S)/guaiacyl (G) ratio decreased, and the enzymatic saccharification yield of glucose from cellulose per fresh weight improved. In these lines, PCW-type CESA genes were upregulated and ferulate 5-hydropxylase1 (F5H1), which is necessary for production of the S unit lignin, was downregulated. In addition, various changes in the expression levels of transcription factors regulating secondary cell wall (SCW) formation were observed. In conclusion, fiber cell-specific ERF041-VP16 improves biomass yield, increases PCW components, and alters lignin composition and deposition and may be suitable for use in future molecular breeding programs of biomass crops.

## Introduction

Advancing technologies that reduce greenhouse gas emissions is an important goal for sustainable development of our society. As the largest terrestrial biomass, lignocellulose has attracted attention in recent decades as a substitute to fossil fuels for the production of energy and chemical substances (FitzPatrick et al., 2010; Anwar et al., 2014). Lignocellulose is an ideal material that does not compete with food supplies unlike other plant materials such as sucrose and starch. Lignocellulosic biomass is primarily composed of cellulose (approximately 40–50%), hemicellulose (approximately 20–30%), and lignin (approximately 10–25%), which are building blocks of the plant cell wall (Anwar et al., 2014; Loqué et al., 2015). All three components exist as polymers in plant cell walls and are associated with each other, resulting in a complex architecture. This complexity interferes degradation of cell-wall constituents by enzymatic and physicochemical approaches. Extraction of each component of lignocellulose with a high degree of purity is required for their use in industrial processes; however, cost-demanding pretreatment of the lignocellulosic biomass hampers industrial use. Thus, an important task for expanding the use of lignocellulosic biomass is the invention of new plants with an ideal cell wall that keeps its stiffness as a timber but enables more efficient isolation of cell wall materials.

To this end, strategies can be roughly categorized into two groups: (1) manipulation of biosynthetic and modifying enzymes of the cell wall components, and (2) introduction or modification of transcription factors (TFs) that broadly control cell wall formation. Lignin, a biopolymer of monolignols, has been studied mainly as a target for modifying cell wall properties. Coniferyl and sinapyl alcohols are major components of monolignols in dicot species and correspond to the lignin polymer guaiacyl (G) and syringyl (S) units, respectively (Whetten and Sederoff, 1995; Vanholme et al., 2012a). Altering the deposition and/or composition of lignin has been demonstrated to affect the enzymatic saccharification yield in a variety of studies using transgenic plants with lignin-modifying genes (Chen and Dixon, 2007; Fu et al., 2011), lignin-deficient mutants (Van Acker et al., 2013), and natural variants (Studer et al., 2011). Moreover, modification of a TF that controls cell wall formation is also an effective way to influence the saccharification yield (Yang et al., 2013; Sakamoto and Mitsuda, 2015; Gui et al., 2020). Activation of a wide range of cell wall-biosynthetic pathways can be more readily achieved by activation or suppression of TF function. We have succeeded in increasing the amount of cell wall components and creating a drastically rigid stem by expressing a TF derived from rice [a homolog of the NAC secondary wall thickening promoting factor (NST) described in detail below] in *Arabidopsis thaliana* and poplar (Sakamoto et al., 2016).

The primary cell wall (PCW) and secondary cell wall (SCW) are the two major structures that form the plant cell wall. The PCW surrounds all plant cells and primarily consists of cellulose, xyloglucan, and pectin, whereas the SCW is mainly composed of cellulose, lignin, and hemicelluloses like xylan and glucomannan depending on species (Albersheim et al., 2010). In the PCW and SCW, different sets of cellulose synthase (CESA) subunits are present in general. In *Arabidopsis*, the PCW-type subunits are CESA1/3/6 (Arioli et al., 1998; Scheible et al., 2001), whereas the SCW-type subunits are CESA4/7/8 (Turner and Somerville, 1997; Taylor et al., 2000). The SCW is formed beneath the PCW in particular tissues, including xylem vessels and fiber cells. Control of SCW formation is achieved through a complex network that involves a number of TFs, and the network structure is a hierarchical cascade (Nakano et al., 2015). The most upstream master regulators are the vascular-related NAC-domain protein (VND) TFs (VND6 and VND7 in *Arabidopsis*) that control SCW formation and programmed cell death in xylem vessels, and the NST TFs [NST1, NST2, and NST3/SCW associated NAC domain protein 1 (SND1) in *Arabidopsis*] that control SCW formation in xylem fibers (Kubo et al., 2005; Mitsuda et al., 2005, 2007; Zhong et al., 2006). Transcription factors MYB46 and MYB83 in *Arabidopsis* are the second-tier master switches, which activate the expression of many of the biosynthetic genes for cellulose, xylan, and lignin that constitute the SCW (Zhong et al., 2007; Ko et al., 2009; McCarthy et al., 2009). Expression of MYB46 and MYB83 is induced by these NAC TFs (Zhong et al., 2007, 2010; McCarthy et al., 2009; Ohashi-Ito et al., 2010; Yamaguchi et al., 2010). Several other NAC, MYB, and other TFs were reported to positively or negatively regulate SCW formation (Nakano et al., 2015).

In previous study, we implemented the new strategy to reconstruct lignocellulose in fibers of the *A. thaliana nst1-1 nst3-1* double knockout mutant that lacks the SCW typically in xylem fibers (Mitsuda et al., 2007; Sakamoto and Mitsuda, 2015). The SCW in xylem vessels is normal in this *A. thaliana* double knockout mutant, and the growth rate is comparable to that of the wild-type *A. thaliana*, whereas the enzymatic saccharification rate is higher than the wild-type plant (Iwase et al., 2009). We searched for factors that synthetically add cell wall components to the xylem fibers of the *nst1-1 nst3-1* mutant. For this purpose, genes encoding chimeric activators and repressors for >300 TFs that are driven by the *NST3* promoter, which predominantly induces expression in the fascicular and interfascicular fibers, were introduced into the *nst1-1 nst3-1* mutant, and the candidates were selected based on the phenotype of the inflorescence stem. In this screening, we identified TFs belonging to the ERFIIId and IIIe subclades (*ERFIIId/e*) of the AP2/ERF family that gave unique traits such as a high enzymatic saccharification rate despite a similar level of cell wall deposition to that of the wild-type plant (Sakamoto et al., 2018). The AP2/ERF family is a plant-specific large TF family involved in various biological phenomena, including ethylene signaling (Fujimoto et al., 2000), wax deposition (Aharoni et al., 2004; Broun et al., 2004), drought response (Liu et al., 1998), callus formation (Iwase et al., 2011), flower architecture (Kunst et al., 1989), root stem cell establishment (Aida et al., 2004), and many others (Nakano et al., 2006). Cell wall analysis of the transgenic lines expressing ERFIIId/e-VP16 in the *nst1-1 nst3-1* mutant strongly suggested that these TF groups have the ability to produce PCW components (Sakamoto et al., 2018). However, the stem strength of the transgenic lines (*NST3p:ERFIIId/e-VP16 nst1-1 nst3-1*) was restored only partially and still showed a pendent phenotype (Sakamoto et al., 2018). On the other hand, constitutive overexpression of *ERF IIId/e* genes by CaMV 35S promoter in wild-type plant induced semi-dwarf phenotype with root growth inhibition (Wilson et al., 1996; Sakamoto et al., 2018; Saelim et al., 2019). From the perspective of industrial applications, enough stem strength and normal growth habit are necessary for the transgenic lines. In this study, we expressed *ERFIIId/e-VP16* transcription factor genes under the control of the *NST3* promoter with the wild-type background to maintain normal stem strength. We observed over-accumulation of the PCW with an unexpected concomitant change of SCW formation. Interestingly, a marked reduction of lignin and its compositional change were found in addition to the expected increase of cell wall residues. Herein, we propose a new strategy to increase cell wall production without increasing its recalcitrance to degradation.

## Materials and Methods

### Plant Materials and Growth Conditions

**Figure 1:**
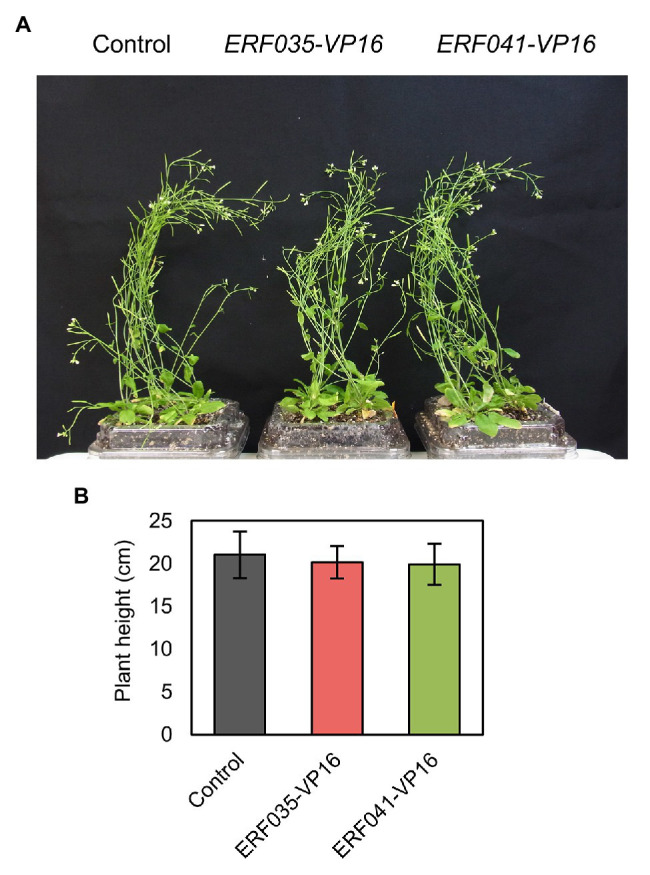
Plant growth of ethylene response factor (ERF) IIId/e-VP16. **(A)** Whole image of the aerial part of T1 plants. **(B)** Plant height of primary stems 42 days after sowing. Data represent mean ± SD.

**Figure 2:**
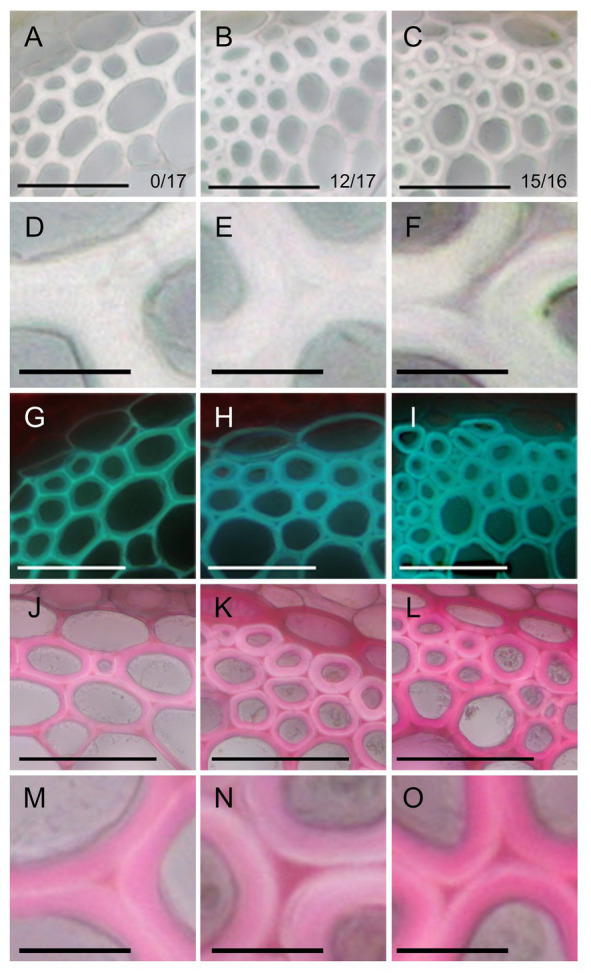
Phenotype of fiber cells of inflorescence stems. Bright field images **(A–F)**, UV autofluorescence images **(G–I)**, and ruthenium red staining **(J–O)** of transverse sections of stems in the control **(A,D,G,J,M)**, ERF035-VP16 **(B,E,H,K,N)**, and ERF041-VP16 **(C,F,I,L,O)** lines. Numbers in **(A–C)** indicate the number of independent lines harboring an altered cell wall from the obtained T1 lines. Bars, 50 μm **(A–C,G–L)**, 10 μm **(D–F,M–O)**.

**Figure 3:**
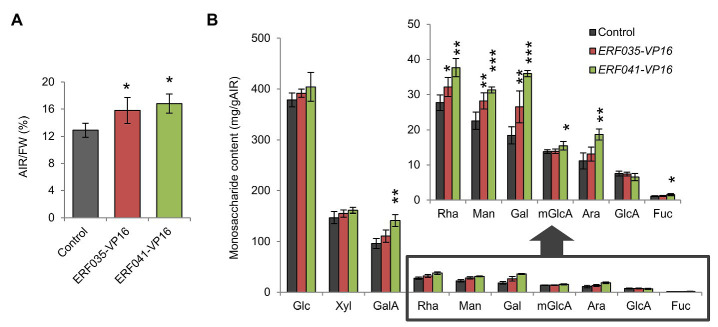
Quantitative analysis of cell wall residues and cell wall-derived monosaccharides. **(A)** Cell wall contents of inflorescence stems as alcohol insoluble residues (AIRs) per fresh weight (FW; AIR/FW). **(B)** Monosaccharide content of individual cell wall samples. Glc, D-glucose; Xyl, D-xylose; GalA, D-galacturonic acid; Rha, L-rhamnose; Man, D-mannose; Gal, D-galactose; mGlcA, 4-*O*-methyl-D-glucuronic acid; Ara, L-arabinose; GlcA, D-glucuronic acid; and Fuc, L-fucose. Data represent mean ± SD performed by biological quadruplicates. ^*^p < 0.05, ^**^p < 0.01, and ^***^p < 0.001 by Dunnett’s test.

**Figure 4:**
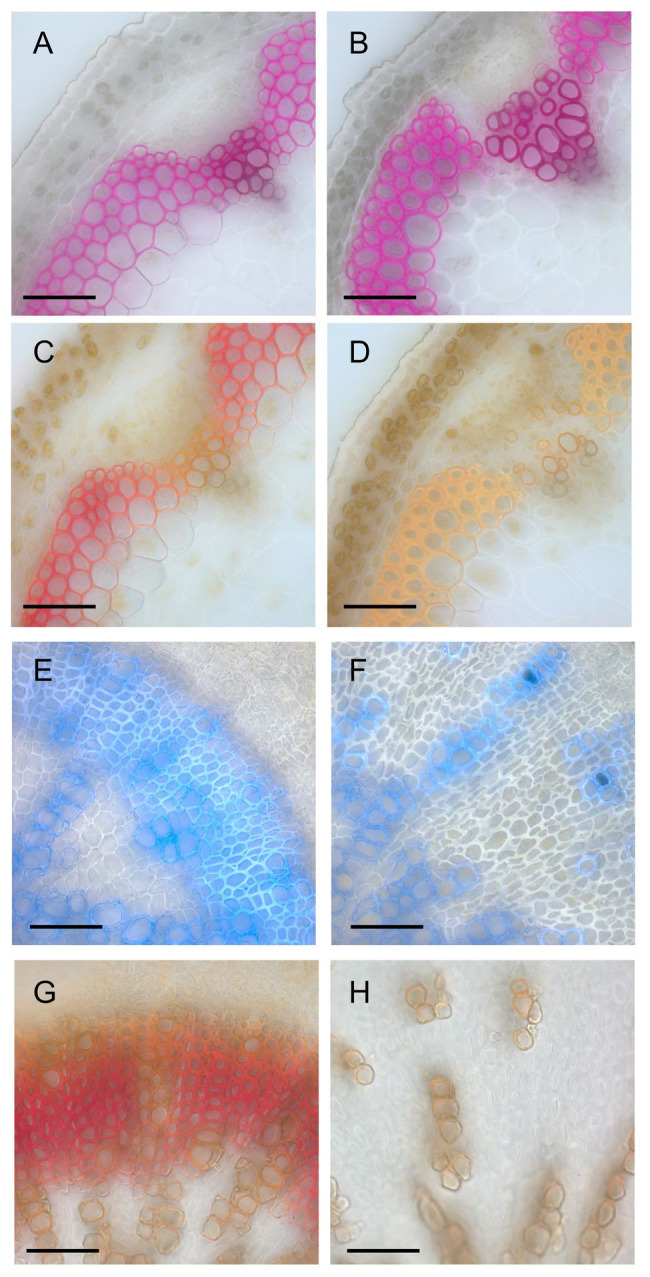
Histological analysis of lignin deposition. **(A,B)** Phloroglucinol-stained sections of the inflorescence stems of wild-type **(A)** and a line (#23) of *ERF041-VP16* **(B)**. **(C,D)** Mäule-stained sections of the inflorescence stems of wild-type **(C)** and #23 of *ERF041-VP16* **(D)**. **(E,F)** Autofluorescence of lignin under UV illumination overlayed onto bright field image for wild-type **(E)** and #23 of *ERF041-VP16* **(F)** hypocotyl sections. **(G,H)** Mäule-stained sections of the hypocotyls of wild-type **(E)** and #23 of *ERF041-VP16* **(F)**. Bars, 50 μm.

**Figure 5:**
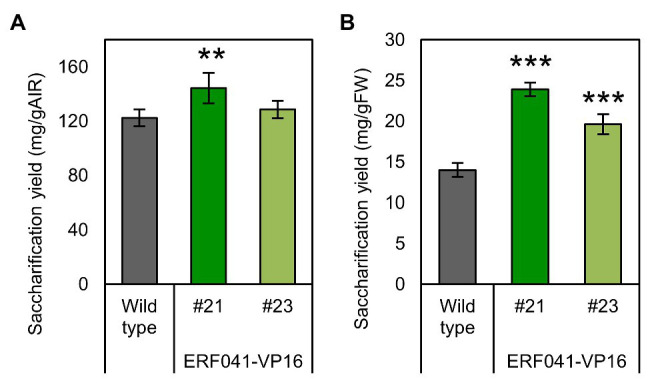
Enzymatic glucose yield of ERF041-VP16. **(A)** Saccharification yields per AIR and **(B)** per FW. Data represent mean ± SD performed by biological quadruplicates. ^**^p < 0.01 and ^***^p < 0.001 by Dunnett’s test.

**Figure 6:**
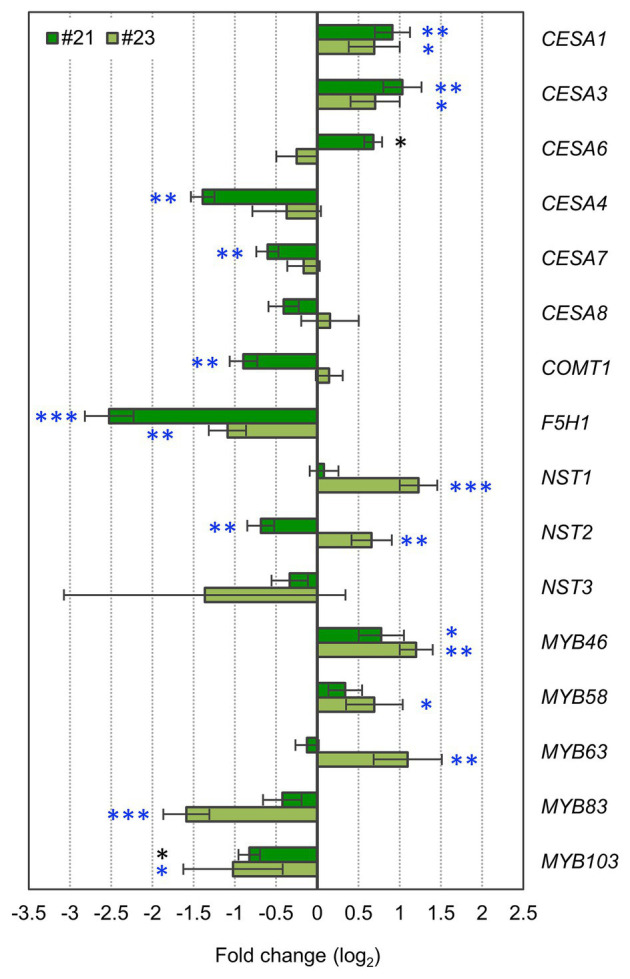
ERF041-VP16 affects primary cell wall (PCW)- and secondary cell wall (SCW)-related genes. Fold changes of PCW- or SCW-related genes. Data represent mean ± SD performed by biological triplicates (*CESA6*, *MYB83*, and *MYB103*) or quadruplicates (the other genes). ^*^p < 0.05, ^**^p < 0.01, and ^***^p < 0.001 by Student’s *t* test. Blue asterisks show significant differences that are also detected using values of *p* adjusted by Benjamini-Hochberg method (*p* < 0.05; Benjamini and Hochberg, 1995). Each expression level was normalized by the normalization factor, estimated from *ACT2*, *PP2AA3*, and *UBQ1* data, and the mean of the wild-type.

*Arabidopsis thaliana* ecotype Columbia-0 (Col-0) was used as the wild-type plant. To generate wild-type-background *NST3pro:ERF035-VP16* and *NST3pro:ERF041-VP16*, previously described vectors (Sakamoto et al., 2018) were introduced into the wild-type plant by *Agrobacterium*-mediated floral dip transformation (Clough and Bent, 1998). “ERF-VP16” represents the ERF protein fused with the transcription activation domain of the herpes simplex virus VP16 protein. The *NST3pro:VAMP722-VP16* line described previously (Sakamoto et al., 2016) was used as a control line. First and second generation transformants (T1 and T2, respectively) were selected on MS plates (1x Murashige and Skoog salt, 0.5% sucrose, 0.5 g/L MES, 0.8% agar, and adjusted to pH 5.7 with KOH) with 30 mg/L hygromycin for 2 weeks and transferred onto soil. Seeds of T3 and wild-type plants were sowed on soil directly. Transgenic plants of the T1 and T2 generations were used for experiments presented in Figures 1–4, respectively. The experiments listed in Table 1 and presented in Figures 5, 6 were performed by using the T3 homo lines of *NST3pro:ERF041-VP16*. Plants were grown under the long-day condition (16 h light, 8 h dark) at 22°C.

**Table 1:** Lignin content and S/G ratio of the inflorescence stems.

		Wild-type	*ERF041-VP16*	
			#21	#23
Lignin (%AIR)		16.59 (0.05)	13.46 (0.33)^***^	15.53 (0.14)^**^
Monomer composition (μmol/g AIR)	S	81.95 (21.53)	13.81 (6.10)^***^	34.93 (4.10)^**^
	G	184.58 (17.08)	143.32 (19.21)^*^	174.38 (11.51)
Monomer composition (μmol/g lignin)	S	494.03 (131.34)	102.29 (43.05)^***^	224.51 (28.87)^**^
	G	1112.46 (105.05)	1069.34 (173.42)	1122.59 (69.03)
S/G ratio		0.45 (0.14)	0.10 (0.03)^***^	0.20 (0.02)^**^

Data represent mean ± *SD* performed by biological quadruplicates.

* *p* < 0.05

** *p* < 0.01

*** *p* < 0.001 by Dunnett’s test.

### Microscopic Observation

Micro-slice (50 μm) transverse sections were prepared as described previously (Sakamoto and Mitsuda, 2015). The 1.5–2.5 cm basal position of the inflorescence stem was embedded in 5% agar, and the agar block was sliced with a vibrating microtome (HM-650V, Thermo Fisher Scientific, Inc., Waltham, MA, United States). The thin sections were fixed in FAA solution (50% ethanol, 10% formaldehyde, and 5% acetic acid). All bright-field and fluorescence images were captured with an Axioskop2 Plus microscope (Zeiss Inc., Oberkochen, Germany) equipped with UV filters (excitation filter: 365-nm short pass; dichroic mirror: 395-nm; emission filter: 400-nm long pass) and AxioCam HRC. For visualization of lignin, staining with phloroglucinol and Mäule reagent was performed as described previously (Yoshida et al., 2013; Sakamoto et al., 2016). For pectin staining, the stem sections were immersed in a 0.02% ruthenium red solution for 1 min. Subsequently, the sections were rinsed with distilled water several times until the supernatant turned transparent. The sections were then placed on a glass slide and observed under a microscope in the bright field.

The width of the cell wall of interfascicular fiber cells was measured in bright-field images of agar sections of the inflorescence stems at the T2 generation with ImageJ/Fiji software (Schindelin et al., 2012). Sample preparation and the procedure for agar sections were described above. Two and three independent lines of ERF035-VP16 and ERF041-VP16, respectively, were selected based on the phenotype reproducibility for this analysis. Three independent *NST3pro:VAMP722-VP16* lines were used as a control. The cell wall between the outermost fiber cells and the adjacent inner cells was used as the measurement site.

### Cell Wall Component Analysis

The 10-cm basal position of the >25-cm inflorescence stem was harvested for cell wall component analysis. Preparation of cell wall residues and monosaccharide composition analysis were performed by methods described previously (Sakamoto et al., 2015; Sakamoto and Mitsuda, 2015). Lignin content was measured using the acetyl bromide method according to Kajita et al. (1997) and Kim et al. (2014). Briefly, 5 mg of alcohol insoluble residue (AIR) material in a glass tube was dissolved in 2.5 ml of 25% (w/w) acetyl bromide in acetic acid. An empty tube was used as the blank. Samples were shaken gently, inverted until the samples were completely dissolved, and incubated at 70°C for 30 min. After cooling on ice, samples were transferred to 50 ml volumetric flasks and 10 ml 2 M NaOH and freshly prepared 0.35 ml 0.5 M (34.75 mg/ml) hydroxylamine hydrochloride were added. The volumetric flasks were then made up to 50 ml with acetic acid, capped and inverted several times to mix. The lignin content was quantified using the known extinction coefficient (23.35 g^−1^ cm^−1^) and absorbance at 280 nm.

For lignin composition analysis, thioacidolysis of plant samples was performed according to the method described by Rolando et al. (1992). Briefly, a freshly made thioacidolysis reagent consisting of 87.5% dioxane, 10% ethanethiol (97%, Alfa Aesar, Haverhill, MA, United States), and 2.5% boron trifluoride diethyl etherate (>47.5% BF3, Sigma-Aldrich, St Louis, MO, United States) by volume were added to a 1 ml screw-cap reaction vial containing AIR material (approximately 5–10 mg). The vial cap was screwed tightly and placed in a heating block at 100°C for 4 h with gentle shaking. After the vial was cooled in ice water for 5 min, 200 μl product mixture solution was transferred into a new vial and 100 μl 1 M sodium hydrogen carbonate was added to adjust the pH to 7. Then, 130 μl 1 M HCl was used to adjust the pH to below 3. The resultant solution was extracted with diethyl ether (250 μl) three times, and the combined organic phase was washed with saturated NaCl and evaporated after drying over anhydrous sodium sulfate. The residues were redissolved in ~250 μl diethyl ether, and 10 μl of the solution was silylated by adding 8 μl N,O-bis(trimethylsilyl)acetamide and incubated at 50°C for 40 min. The resultant solution was analyzed by GC-FID (GC-2010 Plus, Shimadzu Inc., Kyoto, Japan) equipped with a DB-5 capillary column (25 m × 0.25 mm I.D., 0.25 μm film thickness, Agilent Technologies Inc., Santa Clara, CA, United States). The column oven temperature was maintained at 160°C for the first 1 min, and then increased at 10°C/min to 300°C. The split injector (1:10) was maintained at 220°C and the FID was maintained at 300°C. The flow speed of the carrier gas (nitrogen) was 30 cm/s.

Enzymatic saccharification analysis was performed by a slightly modified method described previously (Sakamoto et al., 2015). After cellulase digestion, the reacted suspension was centrifuged at 20,000 x *g* for 5 min. The supernatants were transferred to 0.2 ml PCR tubes and placed for 2 min at 95°C for inactivation of the cellulase. Liberated glucose in the supernatant was determined with the Glucose test kit (FUJIFILM Wako Pure Chemicals Industries, Ltd., Osaka, Japan).

### Gene Expression Analysis

RNA samples were harvested from the 5-cm basal position of 15-cm inflorescence stems (wild-type and transgenic plants of the T3 generation) and immediately soaked in liquid nitrogen. The RNeasy Plant Mini Kit (QIAGEN, Hilden, Germany) was used for extraction of total RNA, and first-strand cDNA was synthesized by the PrimeScript™ RT reagent Kit (TaKaRa, Shiga, Japan). Quantitative PCR was performed using the Power SYBR Green PCR Master Mix or PowerUP SYBR Green PCR Master Mix (Thermo Fisher Scientific) with an ABI 7300 Real-Time PCR system or ABI StepOnePlus Real-Time PCR system. Each gene expression was estimated by standard curve methods and normalized by the geometric mean of the expression level of *ACT2*, *PP2AA3*, and *UBQ1*. Primer sequences are shown in Supplementary Table 1.

## Results

### ERF035-VP16 and ERF041-VP16 Alter Cell Wall Properties of Fiber Cells

In a previous study, we reported that VP16-fused proteins of the AP2/ERF transcription factor family ERFIIId/e (ERFIIId/e-VP16) promote the formation of thickened cell walls with properties of the PCW in the *nst1-1 nst3-1* background (Sakamoto et al., 2018). *ERF035* and *ERF041* belong to the *ERFIIId* and *e* subfamilies, respectively, and their abilities to induce cell wall thickening are relatively high among members of these subfamilies (Sakamoto et al., 2018). In this study, we transformed *NST3pro:ERF035-VP16* (termed “*ERF035-VP16*” hereafter) and *NST3pro:ERF041-VP16* (termed “*ERF041-VP16*” hereafter) into wild-type to investigate whether *ERFIIId/e-VP16* also induces similar cell wall thickening in the wild-type background. Expression of these genes was confirmed not to cause any change to growth habit in wild-type plants (Figure 1A) and there is no statistical difference in the growth of inflorescence stems (Figure 1B).

The phenotype of fibers was observed by taking transverse sections of the inflorescence stem. In the control lines, SCWs spread without gaps among fiber cells (Figures 2A,D). In contrast, the layer between two fiber cells, middle lamella, appeared to be thicker than wild-type and SCWs of two fiber cells were clearly separated by the layer in *ERF035-VP16* (Figures 2B,E) and *ERF041-VP16* (Figures 2C,F). The cell wall of fibers was thicker in *ERF035-VP16* [4.85 ± 0.91 μm (mean ± *SD*); *n* = 299; *p* < 0.001 by Dunnet’s test] and *ERF041-VP16* (7.05 ± 1.13 μm; *n* = 383; *p* < 0.001) when compared with that of the control lines (3.45 ± 0.76 μm; *n* = 319). UV autofluorescence, which reflects the presence of lignin, was stronger in the middle lamella of the control lines (Figure 2G) when compared with the UV positive SCWs of both *ERF035-VP16* and *ERF041-VP16* lines (Figures 2H,I). In addition, ruthenium red-stained pectin was detected predominantly at the corner area in the control lines (Figures 2J,M), whereas pectin staining was detected more widely in the middle lamella of *ERF035-VP16* (Figures 2K,N) and *ERF041-VP16* lines (Figures 2L,O). These phenotypes indicate that *ERF035-VP16* and *ERF041-VP16* have a positive effect on PCW components and a negative effect on lignin.

### ERF035-VP16 and ERF041-VP16 Increase Cell Wall Components Constituting the PCW

ERFIIId/e-VP16s promote the deposition of PCW components in the *nst1-1 nst3-1* background (Sakamoto et al., 2018). To investigate whether ERFIIId/e-VP16s have the same effect in the wild-type background, the cell wall components were isolated from the inflorescence stem of the wild-type-background *ERF035-VP16* and *ERF041-VP16* lines, and the amount of cell wall residue and monosaccharides present in the cell wall was measured. The AIR per fresh weight (AIR/FW) corresponding to the cell wall amount of *ERF035-VP16* and *ERF041-VP16* was 1.2-fold higher than the control lines, and this difference was significant (Figure 3A). Among the monosaccharides isolated from samples digested completely with concentrated sulfuric acid and heat, L-rhamnose (Rha), D-mannose (Man), and D-galactose (Gal) amounts increased significantly in both transgenic lines. In addition, the amounts of D-galacturonic acid (GalA), 4-*O*-methyl-D-glucuronic acid (mGlcA), L-arabinose (Ara), and L-fucose (Fuc) were significantly higher in *ERF041-VP16* than in the control line (Figure 3B). Rha and GalA are major components of pectin, and Fuc is derived from xyloglucan. Therefore, these data showed that *ERF035-VP16* and *ERF041-VP16* also increased the deposition of cell wall components predominantly found in the PCW in a wild-type background, which is in agreement with that observed previously for the *nst1-1 nst3-1* background (Sakamoto et al., 2018). The results also indicated that *ERF041-VP16* has a severer effect than *ERF035-VP16*.

### ERF041-VP16 Alters Lignin Deposition and Composition

Results presented in Figure 2 showed that the intensity of UV autofluorescence decreased in the middle lamella of interfascicular fiber cells of *ERF041-VP16*. For a deeper understanding of the change in lignin, we performed phloroglucinol staining and Mäule staining of inflorescence stems. In wild-type, typical red staining by phloroglucinol was observed in interfascicular fibers, vessels, and fascicular fibers (Figure 4A), and red purple staining by the Mäule stain was detected in all phloroglucinol-stained cells (Figure 4C). In contrast, in *ERF041-VP16*, a similar staining pattern of phloroglucinol to that observed in wild-type was detected in the interfascicular fibers and vessels, but the staining was weak or absent in fibers from the fascicular region (Figure 4B, Supplementary Figure 1A). Interfascicular fibers stained with the Mäule stain were orange in *ERF041-VP16*, which makes a striking contrast to the wild-type results (Figure 4D, Supplementary Figure 1B). The hypocotyl section was also investigated to further understand this observation. The wild-type hypocotyl exhibits secondary growth after flowering. Well-developed fibers emitted blue autofluorescence of lignin under UV illumination (Figure 4E) and showed red coloration by Mäule staining (Figure 4G). However, in the hypocotyl of *ERF041-VP16*, the xylem cells, except for sparsely embedded vessels, showed weak or no autofluorescence of lignin and staining by Mäule reagent even though clear thickness of cell wall was observed (Figures 4F,H, Supplementary Figures 1C,D). These results indicated that *ERF041-VP16* inhibits the normal deposition of lignin in general and may also affect the S/G ratio of lignin of the cell wall in the interfascicular region.

We performed biochemical assays of lignin to further clarify these results. Two of the T3-homo lines of *ERF041-VP16*, #21 and #23, were selected based on phenotypic reproducibility by microscopic observation. The over-accumulation of *ERF041* transcripts, which was derived from *ERF041-VP16*, was confirmed by quantitative RT-PCR analysis (1,179-fold and 144-fold higher in lines #21 and #23, respectively, when compared with that of wild-type). We then determined the total lignin content, S and G units of lignin, and the S/G ratio. In the #21 and #23 lines, the total amount of lignin was reduced significantly to 81 and 94% of the wild-type, respectively (Table 1). In the higher-expression line #21, S and G units were reduced to 17 and 78% of the wild-type, respectively, and these changes were significant (Table 1). In the moderate-expression line #23, the S unit was reduced significantly to 43%, but no significant change in the G unit content was detected (Table 1). The S/G ratio of both lines was significantly lower than that of the wild-type (Table 1). In summary, these results strongly indicate that *ERF041-VP16* suppresses lignin deposition, especially S units, resulting in a change in lignin composition.

A decrease in lignin deposition has been reported to have a positive effect on saccharification yields by cellulase treatment (Chen and Dixon, 2007). We investigated saccharification yields per AIR from inflorescence stems of the two *ERF041-VP16* lines. In the higher-expression line #21, the saccharification yield increased significantly, whereas no significant change was detected in the moderate-expression line #23 (Figure 5A). Furthermore, saccharification yields per fresh weight were 1.71-fold and 1.40-fold higher than that of the wild-type in lines #21 and #23, respectively (Figure 5B). *ERF041-VP16* improves the saccharification yield per fresh weight mainly by increasing the ratio of AIR, but in the line where the lignin content is sufficiently reduced, higher cell-wall digestibility may also contribute to the improvement.

### ERF041-VP16 Induces Expression of PCW-Type CESAs and Suppresses F5H1 Expression

Our results suggest that ERF041-VP16 activates thickening of the PCW in the wild-type background. Expression levels of PCW-type *CESA*s, i.e., *CESA1/3/6*, were reported to increase in stems of *nst1 nst3*-background *NST3pro:ERFIIId/e-VP16*, *ERFIIId/e*-expressed protoplasts, and estrogen-induced *ERFIIId* overexpression lines (Sakamoto et al., 2018; Saelim et al., 2019). Our quantitative PCR analyses showed that expression levels of *CESA1* and *CESA3* also increased significantly in the wild-type-background *ERF041-VP16* (Figure 6). In addition, the expression level of the *CESA6* gene showed a significant increase in line #21 (Figure 6). Among the SCW-type *CESA* genes, i.e., *CESA4*/*7*/*8*, expression levels of *CESA4* and *CESA7* decreased significantly in the higher-expression line #21 but did not change in the moderate-expression line #23 (Figure 6).

According to our data, *ERF041-VP16* suppresses the deposition of the S unit of lignin. The S unit and the G unit of lignin are derived from sinapyl alcohol and coniferyl alcohol, respectively. Ferulic acid 5-hydroxylase (F5H) and caffeic acid/5-hydroxyferulic acid *O*-methyltransferase (COMT) mediate the reaction from coniferaldehyde, the precursor of coniferyl alcohol, to sinapaldehyde, the precursor of sinapyl alcohol. The *F5H1* gene and the *COMT1* gene are essential for the deposition of the S unit of lignin (Meyer et al., 1998; Franke et al., 2000; Goujon et al., 2003; Weng et al., 2010; Vanholme et al., 2012b). In both lines of *ERF041-VP16*, a significant reduction in the expression level of *F5H1* to less than half that of the wild-type was observed (Figure 6). In the higher-expression line #21, the expression of *COMT1* was also reduced by approximately 50% when compared with that of the wild-type, whereas no significant change in the moderate-expression line #23 was observed (Figure 6). These results suggest that the reduced levels of the S unit of lignin in *ERF041-VP16* were caused primarily by the downregulation of the *F5H1* gene.

Next, we investigated the expression levels of eight SCW-regulatory genes (*NST1*, *NST2*, *NST3*, *MYB46*, *MYB58*, *MYB63*, *MYB83*, and *MYB103*). SCW-regulatory genes showed inconsistent changes between two lines of *ERF041-VP16*. In the higher-expression line #21, *MYB46* was upregulated but *NST2* and *MYB103* were downregulated significantly (Figure 6). In the moderate-expression line #23, *NST1*, *NST2*, *MYB46*, *MYB58*, and *MYB63* were upregulated but *MYB83* and *MYB103* were downregulated (Figure 6). Only *MYB46* and *MYB103* showed common changes between the two lines.

In summary, the results showed that *ERF041-VP16* commonly upregulates the expression of PCW-type *CESA*s and downregulates the expression of *F5H1*. The expression of SCW-regulatory genes may be affected differently depending on the expression level of *ERF041-VP16*.

## Discussion

In a previous report, we successfully replaced the SCWs of fibers with cell wall components similar to those found in the PCW by a combination of *nst1 nst3* mutations and *ERFIIId/e-VP16* (Sakamoto et al., 2018). In this study, we clarified that fiber cell-specific expression of *ERFIIId/e-VP16*, in particular *ERF041-VP16*, improves biomass yield in the wild-type background. Fiber-specific expression of *ERFIIId/e-VP16* did not induce any growth defects, such as constitutive expression to yield a dwarf phenotype or root growth inhibition (Wilson et al., 1996; Sakamoto et al., 2018; Saelim et al., 2019). Histological analysis showed that *ERFIIId/e-VP16* promotes cell wall thickening and over-accumulation of cell wall components, including pectin in the area outside the SCW. Furthermore, the monosaccharide assay revealed over-accumulation of PCW components, which may be the major cause of the increased biomass yield. We confirmed that PCW-type CESAs were upregulated by fiber-specific *ERF041-VP16* even in the wild-type background and as observed in the *nst1 nst3* background or as in the case of constitutive expression (Sakamoto et al., 2018; Saelim et al., 2019). Importantly, saccharification yield by cellulase treatment per fresh weight was higher than the wild-type. Our strategy is easily applicable to industrial crops because *ERF041-VP16* can be used in the wild-type background.

In addition to improved biomass yield, we found that *ERF035-VP16* and *ERF041-VP16* altered lignin properties. *ERF041-VP16* suppressed lignin deposition, especially in the fascicular region of the inflorescence stems. In two independent lines of *ERF041-VP16*, total lignin and the S unit of lignin were reduced significantly. In the higher-expression line #21, the amount of the G unit in lignin also decreased significantly and the saccharification yield increased, whereas in the moderate-expression line #23, the amount of G unit did not change and no significant increase in the saccharification yield was observed. The *F5H1* gene was downregulated in the two independent *ERF041-VP16* lines. *COMT1* was also downregulated in the higher-expression line #21. Therefore, we concluded that downregulation of *F5H1* is the major cause of the reduced amount of the S unit, and the downregulation of *COMT1* also plays a role. Because ERF IIId/e transcription factors are transcriptional activators (Sakamoto et al., 2018; Saelim et al., 2019) and the transcriptional activation domain, VP16, further enhances transcriptional activation capability (Fujiwara et al., 2014a,b), the downregulation of *F5H1* and *COMT1* must be indirectly triggered. Moreover, in the higher-expression line #21, lignin deposition was drastically inhibited and an improvement in the saccharification yield was observed. Because a loss-of-function mutant of *F5H1* (*fah1*) and *COMT1* (*Atomt1*) did not caused a decrease in lignin content (Meyer et al., 1998; Goujon et al., 2003), there must be other factors responsible for the observed decrease in the total lignin content. Since carbon supply is limited, increased production of PCW components may indirectly suppress lignin biosynthesis. The result of monosaccharide composition analysis (PCW-related sugars were increased but SCW-related sugars were unchanged) supports the hypothesis that there is such a balancing effect between PCW components and lignin. Although some points remain to be clarified, a plant with appropriate lignin content and better saccharification yield should be obtained through proper selection from some *ERF041-VP16* lines.

In our analyses, expression changes of SCW-related genes did not show consistent results between the two *ERF041-VP16* lines. Interestingly, expression of SCW-type CESA genes decreased in the higher-expression line #21. In *A. thaliana* and rice, *ERFIIId* genes, including *ERF035*, are co-expressed with SCW-related genes (Saelim et al., 2019). Overexpression of the *ERFIIId* genes by more than 50-fold when compared with that of wild-type caused a decrease in the expression of SCW-type CESAs (Saelim et al., 2019), which is consistent with our results for *ERF041-VP16*. Additionally, in the moderate-expression line #23, SCW-regulatory transcription factors were generally upregulated, except for *MYB83* and *MYB103*. The regulatory network of the SCW consists of a large number of components and complex interactions (Taylor-Teeples et al., 2015). Our results suggest that the SCW regulatory network has multiple different stable states that are dependent on the expression level of *ERF041-VP16*. Taken together, these findings indicate that *ERFIIId/e* genes affect the SCW pathway through activation of some factor(s), but their effects vary and are dependent on their expression levels. To clarify how the downstream pathway is affected by the expression levels of *ERFIIId/e* and which factors mediate control, comprehensive genetic, and/or biochemical analysis are/is required.

According to Loqué et al. (2015), there are the following plant biotechnological approaches to address the recalcitrance of lignocellulosic biomass: (1) reduce or alter the lignin polymer network; (2) reduce processing inhibitors derived from the plant biomass; and (3) increase the abundance of fermentable sugars (e.g., cellulose and hemicellulose). As genetic modifications that simultaneously satisfy the first and the third approaches, coordinated changes to lignin and cellulose/hemicellulose have been reported (Hu et al., 1999; Li et al., 2003; Ambavaram et al., 2010; Vermerris et al., 2010). Downregulation of *4-coumarate:coenzyme A ligase* in transgenic aspen (*Populus tremuloides*) trees increased and decreased the abundance of cellulose and lignin, respectively (Hu et al., 1999; Li et al., 2003). Additional expression of *CALd5H* in these transgenic trees increased the S/G ratio and caused further changes to lignin and cellulose levels (Li et al., 2003). Maize brown midrib mutants displayed reduced content of lignin and increased content of hemicellulose (Vermerris et al., 2010). Overexpression of the *A. thaliana SHINE* transcription factor (*AtSHN*) gene in rice caused a 34% increase in cellulose, 45% reduction in lignin content and improved digestibility (Ambavaram et al., 2010). This gene has been suggested to be involved in biosynthesis of both lignin and cellulose *via* control of *NST1*/*2*/*3*, *VND6*, and *MYB* transcription factor genes in rice (Ambavaram et al., 2010). Its overexpression in *A. thaliana* altered expression of biosynthetic pathway genes of lignin and cellulose/hemicellulose but caused no visible change in cross sections (Ambavaram et al., 2010). Because *ERF041-VP16* affects both the PCW component and lignin cooperatively, *ERF041-VP16* represents a potentially effective method for improving saccharification yields following investigation of its applicability to trees and crops. However, how the balance among PCW, SCW, and other types of cell walls (e.g., the G layer formed in tension wood) is genetically controlled and what is the ideal proportion of them remains unresolved. Given the versatile function of ERFIIId/e in PCW thickening, revealing how ERFIIId/e affects the lignin pathway should provide insights into the regulatory mechanism for balancing different types of cell walls in plant.

## Data Availability Statement

The original contributions presented in the study are included in the article/Supplementary Material, further inquiries can be directed to the corresponding author.

## Author Contributions

MN, SS, and NM contributed to the conception of the study. SS, Nuoendagula, and MN performed the analysis of the cell wall components, lignin composition, and other parts, respectively. SK supervised the analysis of lignin composition. MN wrote the initial draft of the manuscript. All authors contributed to the interpretation of data, manuscript revisions, and read and approved the submitted version.

### Conflict of Interest

The authors declare that the research was conducted in the absence of any commercial or financial relationships that could be construed as a potential conflict of interest.
