# Supplementary figures and images for: Fiber Cell-Specific Expression of the VP16-Fused Ethylene Response Factor 41 Protein Increases Biomass Yield and Alters Lignin Composition
Source: Front Plant Sci. 2021 Apr 30;12:654655. doi: 10.3389/fpls.2021.654655 (PMC8121085; doi:10.3389/fpls.2021.654655)

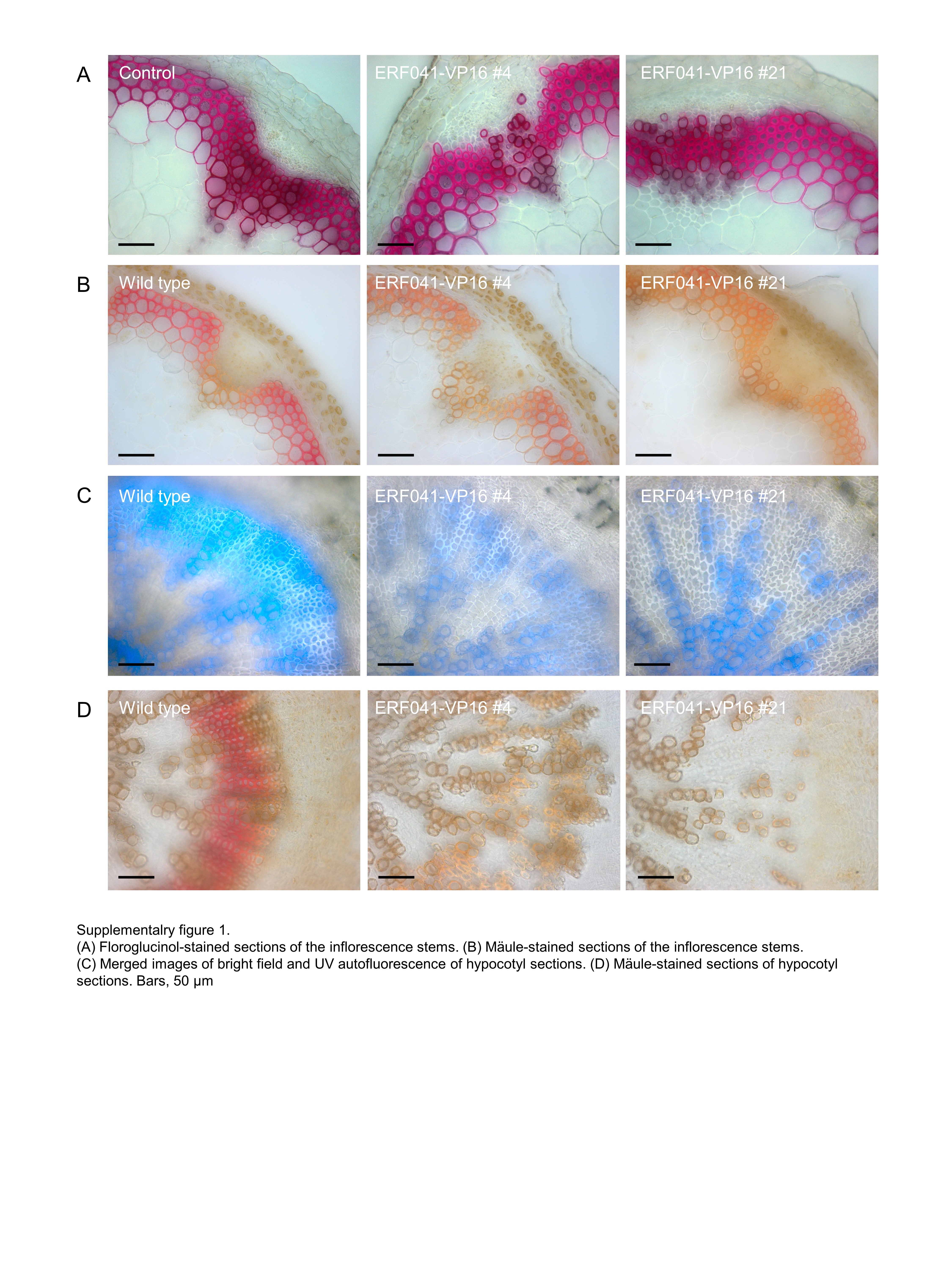

Supplement: Supplementary file 1 [file Image_1.JPEG]
