# Supplementary material for: Fiber Cell-Specific Expression of the VP16-Fused Ethylene Response Factor 41 Protein Increases Biomass Yield and Alters Lignin Composition
Source: Front Plant Sci. 2021 Apr 30;12:654655. doi: 10.3389/fpls.2021.654655 (PMC8121085; doi:10.3389/fpls.2021.654655)
Supplement: Supplementary file 2 [file Table_1.pdf]

Supplemental table 1. Primer list

Gene name	Primer F	Primer R
PP2AA3	GACCAAGTGAACCAGGTTATTGG	TACTCTCCAGTGCCTGTCTTCA
ACT2	GACCAGCTCTTCCATCGAGAAG	AGCAGCTTCCATTCCCACAAAC
UBQ1	TGAGCCTTCCTTGATGATGCT	GCACTTGCGGCAAATCATCT
ERF041	CCCTCGACCCGTTTCATTAAG	TCGGTTCCATGTGAGCTGCTTTA
CESA1	TGTCTCATCACCGACAGATTCATC	CATCTCAGCTCCAGGATTCCA
CESA3	CTCGATGACATAGAAGAGGGAGTT	CCAGGCTCATTTGCGACATTAAGA
CESA6	CTCTCTACCGCTCATCGTTTAC	GACGAGAAGAGCGCCATGAA
CESA4	CGGCAAGTTCATCATTCCTACGA	CACTCCACCGGAGTTCTAAGA
CESA7	TGGAATTGAGATGGAGCGGAGTTA	AGACCTTGGACAACCGCAAAGA
CESA8	GTCACATCGAAAACCGCAGATGAT	AAGTGACGTCGGAGGGATCAA
COMT1	ATGCTCCTTCTCATCCTGGTATTG	GCAATGTTCGTCACTCCAGTCA
F5H1	GTCAGTGAGACAGCGGATCTT	CCGTTTCCGTTCCTCCAAACA
NST1	ACCGTTCATGAGGTCGTGAGT	TCCTCCGACGGGACTGTTTA
NST2	ACCGGTCCGGTTGTAATACC	CACTTAGTCACTTCCTCTCTTCCA
NST3	GCCTTGGGATATTCAAGAGGAATG	ACTTCTTGTCCTTGTGGCTGAAGA
MYB46	GGACAAGGATGTTGGAGTGATG	GGACGAAGATAGTTGATCCAACGA
MYB58	TCTCCCAAAGCAAGCTGGATTG	GTGTCTTCTTCCTCTGCACTGA
MYB63	TCACCACAACTATGGGAACAAGTG	GATGAGTGTGCCACACATTCTTGA
MYB83	TCGCCTTCGCTGGATCAATTAC	GCTATTTGAGACCACCTGTTACCA
MYB103	TGGAGTTGTGGGAAACAGGTG	AGGCCTCGATGGTAGTGGCA
